# Corticosteroids effects on LPS-induced rat inflammatory keratocyte cell model

**DOI:** 10.1371/journal.pone.0176639

**Published:** 2017-04-27

**Authors:** Huize Yan, Yingwei Wang, Shuhao Shen, Zheng Wu, Pengxia Wan

**Affiliations:** 1 Department of Developmental and Regenerative Biology, Jinan University, Guangzhou, China; 2 Key laboratory for Regenerative Medicine, Ministry of Education, Jinan University, Guangzhou, China; 3 Department of Ophthalmology, First Affiliated Hospital of Jinan University, Guangzhou, China; 4 Ophthalmology Department, First Affiliated Hospital of Sun Yat-sen University, Guangzhou, China; Wayne State University, UNITED STATES

## Abstract

**Purpose:**

Corticosteroids are efficient anti-inflammation treatments. However, there are still arguments on whether it should be used in keratitis. This study was to observe the effect of corticosteroids on keratocytes both in normal condition and inflammation status *in vitro*.

**Methods:**

Rat keratocytes were cultured and used for examination. 10 μg/ml lipopolysaccharide (LPS) was used to establish the inflammatory keratocyte cell model, and prednisolone acetate (PA), dexamethasone (Dex) and fluorometholone (Flu) were used as corticosteroids treatments. 5 d-growth curve and cell viabilities were assayed by CCK8, and cell morphologies and migration rate were studied. TNF-α, IL-6 and IL-1β levels were examined by ELISA. Western blotting was used to quantified type VI collagen (Col VI) and matrix metalloproteinase 9 (MMP9) expressions, and immunofluorescence staining assays of Col I and Col VI were carried out.

**Results:**

In normal condition, proliferation and migration of keratocytes were slightly influenced in PA, Dex and Flu groups. The secretion of Col I and Col VI was suppressed and MMP9 expression increased in corticosteroids groups. But no significant difference was seen in TNF-α, IL-6 and IL-1β expression levels. In inflammatory status, TNF-α, IL-6 and MMP9 levels increased in LPS group, while they significantly decreased in corticosteroids groups. Although keratocytes viabilities and migration were slightly affected in 24 h, no significant differences were seen between LPS group and corticosteroids groups in 5-d proliferation. Col I and Col VI secretion in LPS-keratocytes was maintained with corticosteroids treatments.

**Conclusions:**

Corticosteroids showed lightly effects on keratocytes proliferation and migration, but it successfully decreased TNF-α, IL-6 level and maintained the secretion of and Col I and Col VI, while suppressed the expression of MMP9 in LPS-induced keratocytes. PA was suggested to use in early stage of keratitis clinical treatment.

## Introduction

Around 5 million corneal blindness patients have been reported in China, and infectious keratitis is the most common causes for this devastating number. During keratitis, activated inflammatory cells like neutrophil would release series of inflammatory cytokines, such as tumor necrosis factor-alpha (TNF-α), interleukin-6 (IL-6) and interleukin-1beta (IL-1β),[1] which later induce keratocytes necrosis and collagen degradation in stroma.[2, 3] These activities could result in the thinning of cornea stroma and subsequent scarring formation. Thus, it is of great importance to inhibit the inflammatory cascade in the early stage of keratitis.[4]

Corticosteroids eye drops, such as prednisolone acetate (PA), dexamethasone (Dex) and fluorometholone (Flu), are common-used anti-inflammatory drugs during clinical treatments. Previous studies demonstrated that corticosteroids like Dex phosphate contributed to an anti-inflammatory effect through inhibiting the nuclear factor-kappa B (NF-κB) signal pathway.[5, 6] However, whether corticosteroids eye drops are proper for infectious keratitis cure remains a controversial topic.[7] Lu et al. proved that dexamethone could inhibit IL-1-induced collagen degradation and decrease stroma scarring.[3] On the other hand, corticosteroids may have pro-apoptotic effect on corneal epithelial cells and keratocytes,[8] which may worsen corneal stroma ulcer during keratitis. Although several clinical studies showed that additional use of corticosteroids at the early stage of bacterial keratitis could be beneficial,[7, 9] still some data demonstrated that no significant cure effects were observed.[10] Therefore, it is important to understand the effect of corticosteroids on keratocytes in normal and inflammatory situation.

By studying the effect of typical corticosteroids eye drops main components, PA, Dex and Flu, on rat keratocytes *in vitro*, we could explore the compatibility of corticosteroids in treating keratitis. Moreover, it was reported that 19% of the infectious cases were produced by bacterial keratitis, of which 26% (resulted from wearing contact lens) were caused by gram-negative bacteria.[11, 12] Since LPS was the main component of endotoxin from gram-negative bacteria, it was reasonable to use it for inflammatory keratocyte cell model establishment. Therefore, the present study would focus on the effect of corticosteroids on keratocytes both in normal and inflammatory condition, and cell bioactivities such as proliferation, migration and collagen secretion would be studied.

## Materials and methods

### Isolation and culture of keratocytes

Male Sprague Dawley rats, aged 3 weeks, were purchased from Guangdong Medical Experimental Animal Center. All experiments were carried out in accordance with the ethical guidelines of National Guide for the Care and Use of Laboratory Animals and approved by Jinan University Animal Care and Use Committee. After being humanely euthanized, eyeballs were collected and then thoroughly washed with phosphate buffer saline (PBS). Then the eyeballs were immersed in 10 mg/ml dispase II in Dulbecco Modified Eagle Medium (DMEM) overnight at 4°C. Corneal stroma was separated from eyeballs after removing epithelial and endothelial layer. The stroma was cut into pieces and immersed in 0.1% type IV collagenase (life technologies, NY, USA) for 15 min at 37°C before centrifugation. The isolated tissues were cultured in DMEM contained 0.1% insulin (Biosharp, Anhui, China), 0.1% transferring (Sigma-aldrich, MO, USA), 0.1% epidermal growth factor (EGF, Pepro tech, NJ, USA), 1% none-essential amino acid (NEAA, Corning, VA, USA), 1% L-glutamine (Corning, VA, USA), 1% penicillin-streptomycin and 10% FBS(BD Bioscience, NJ, USA) at 37°C under a humidified atmosphere of 5% CO_2_. After expanded and passaged, P2~P5 cells were used for experiments.

### *In vitro* keratitis stroma cell model

To observe the effect of corticosteroids on keratocytes both in normal condition and under keratitis, LPS was used to establish an inflammatory keratocyte cell model *in vitro*, and typical corticosteroids eye drops main components (PA, Dex and Flu) were used as treatments. Control group, PA group, Dex group, Flu group, LPS group, -PA-LPS group,—Dex-LPS group and Flu-LPS group were set. 10 μg/ml LPS were used to induce inflammatory keratocyte cell model. PA, Dex and Flu (Sigma, MO, USA), were dissolved in ethyl alcohol, at a final working concentration of 10^−9^ mmol/ml, and PBS was used in the control group.

### *In vitro* cell migration assay

1.5×10^5^ keratocytes were cultured in 12-well plates to create a confluent monolayer, and a straight linear wound was made by using a sterile 1000 μl pipette tip to scratch perpendicular to the cell layer. Each well was rinsed twice gently with PBS in order to remove detached cells and debris. After that, medium containing 10^−9^ mmol/ml corticosteroid solution of PA, Dex orFlu were added correspondingly in each group, and 10 μg/ml LPS were added in the four LPS groups. The size of wounds were observed and measured at 0 h, 6 h, 12 h and 24 h after scratching. For each image, distance between both sides of the scratch was measured by Image Pro-Plus software (Media Cybernetics), and the migration rate at different time was calculated as the following formula: (0 h distance—set time distance)/ time (Pixel/h).

### Cell viability assay

Cell Counting Kit-8 (CCK8, WST, China) was used for cell viability assay. In brief, rat keratocytes were seeded in a 96-well plate at the density of 1000 cells/100 μl medium/well. Mediums were replaced every day after the test. Different mediums containing corticosteroids with/without LPS were given at the beginning of test. Cell viabilities were determined at the first 12 h and every 24 h during the following 5 days. 10 μl CCK8 was added to each well and incubated for 1 h, and the optical density (OD) value at 450 nm wavelengths was determined by microplate reader (Synergy, USA). Cell morphologies in different groups were recorded. Also, the short-term effects of corticosteroids on normal and LPS-condition were tested through the same method. Keratocytes were seeded 5000 cells/well in a 96-well plate, and the OD values were tested at 0 h, 6 h, 12 h and 24 h.

### Immunofluorescence staining

To determine the expression of Col I, VI under the effect of corticosteroids, 5×10^3^ cells were seeded onto glass slides. After treated with different mediums for 48 h, cells were fixed by 75% absolute ethyl alcohol for 10 min. Then slides were washed with PBS for 3 times, 10 min each. The slides were incubated in 0.1% Triton X-100 in PBS for 30 min before washed for 3 times and then incubated in Goat serum (Sigma, USA) for 1 hour. Then a mouse anti-type I collagen antibody (Abcam, UK, 1:200), or a rabbit anti-type VI collagen antibody (Abcam, UK, 1:200) was added and incubated at 4°Covernight. After washed with PBS, a goat anti-mouse 488 secondary antibody(Abcam, UK, 1:200), or a goat anti-rabbit IgG Cy3 secondary antibody (Abcam, UK, 1:200) was added and incubated for 2 h at room temperature. The nuclei were counterstained with Hoechst 33528 (Invitrogen, USA, 1:1000). Results were observed and recorded by laser confocal scanning microscope (Nikon, Japan) and analyzed with a Nikon vision imaging system.

### Western blot analysis

Keratocytes were cultured in 6 wells plate (5x10^5^ cells/well). After incubated with different treatments for 48 h, total protein was extracted from the lysed sample in RIPA buffer, and protein levels were quantified by protein quantitative analysis kit (Bocai, China). Samples were run on 8% SDS-PAGE gels for 2 hours at 110 V before transferred to a polyvinylidene fluoride (PVDF) membrane (Millipore, USA). After blocked in 5% bovine serum albumin in TBST for at least 1 hour, that bands were incubated with the following primary antibodies: rabbit monoclonal antibody against type VI collagen (Abcam, UK), rabbit polyclonal antibody against matrix metalloproteinase 9 (Proteintech, USA) and rabbit monoclonal antibody against GAPDH (Cell Signaling Technology, USA) in TBST overnight at 4°C. Washed 3 times with TBST, the blots were then incubated with HRP-linked secondary antibody anti-rabbit IgG (Cell Signaling Technology, USA). The results were visualized via enzyme-linked chemiluminescence by ECL kit (Thermo, USA). The densitometry of the immunoreactive bands was measured with Image J software. GAPDH was regarded as an internal control. The relative expression of Col VI and MMP9 in each group was calculated by dividing the gray value of Col VI (MMP9) to GAPDH.

### Enzyme-linked immunosorbent assay (ELISA)

To determine the inflammatory response in each group, the levels of TNF-α, IL-6 and IL-1β level were evaluated. After cells incubated with different mediums for 48 h, supernatant of each group was collected and stored at -30°C before assay. The quantification of TNF-α, IL-6 and IL-1β was based on a quantitative sandwich immunoassay with ELISA detection kits for rat (Neobioscience, China). All procedures were conducted according to the manufacturer’s instructions. Absorbance was read at 450 nm wavelengths using a microplate reader (Synergy, USA).

### Statistical analysis

Data were expressed as mean ± standard deviation, and all results were repeated at least 3 times. The difference between 2 groups was analyzed using one way ANOVA with SigmaPlot11.0 software (GmbH, Germany). *p* value<0.05 was considered as significant difference.

## Results

### Effects of corticosteroids on keratocytes bioactivities in normal condition

#### Corticosteroids affected keratocyte proliferation, migration and viability

**Fig 1:**
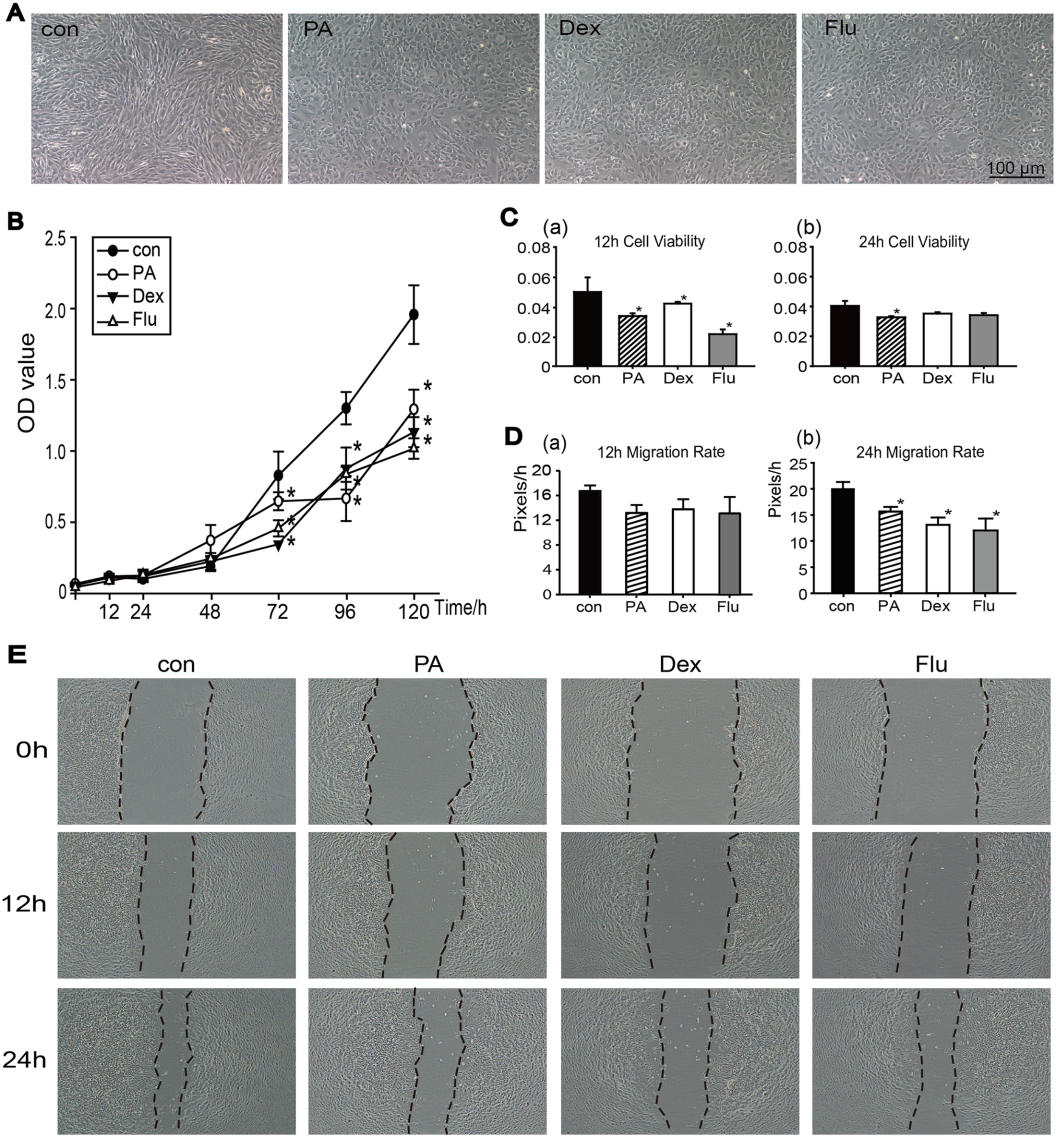
Corticosteroids effects on keratocytes under normal culture conditions. (A)Morphological results of keratocytes after 5-d treatments with PBS, PA, Dex and Flu. (B)Proliferation in each group was determined by CCK8 assayed at 12 h, and then every 24 h during 5 days. (C)Short-term cell viabilities in each group were tested by CCK8 at 12 h (a) and 24 h (b). (D)The migration rates of keratocytes at 12 h(a) and 24 h(b) were evaluated by Image Pro-Plus software (Media Cybernetics) using the following formula: (0 h distance—set time distance)/ time (Pixel/h). (E)Migration abilities of keratocytes grown in normal condition with corticosteroid treatments at 0 h, 12 h and 24 h. Data were analyzed by one way ANOVA,*p<0.05 versus control group (n = 3).

Rat keratocytes showed polygon morphology in three corticosteroids groups, compared to the long spindle shaped of control group (Fig 1A). In the early 48 h of growth curve assay, no significant differences between control group and three corticosteroid groups were observed. However, the effect of corticosteroids revealed at day 3, and PA, Dex, Flu showed suppression on keratocytes proliferation compared to the control (Fig 1B). Additionally, in the 24 h short-term cell viability assay, all three corticosteroids treatment groups showed suppression effect at 12 h (Fig 1C(a)), but no significant differences were observed at 24 h assay in the Dex and Flu groups compared to control group (Fig 1C(b)). An *in vitro* cell migration assay was performed to determine keratocytes migration abilities in normal condition (Fig 1D and 1E). The use of PA, Dex and Flu showed suppression on keratocytes migration abilities at 12 h and 24 h compared to control group, but statistic significance was only observed at 24 h (Fig 1D; *p*<0.05, n = 3).

#### PA showed no suppression effect on collagen expression in normal condition

**Fig 2:**
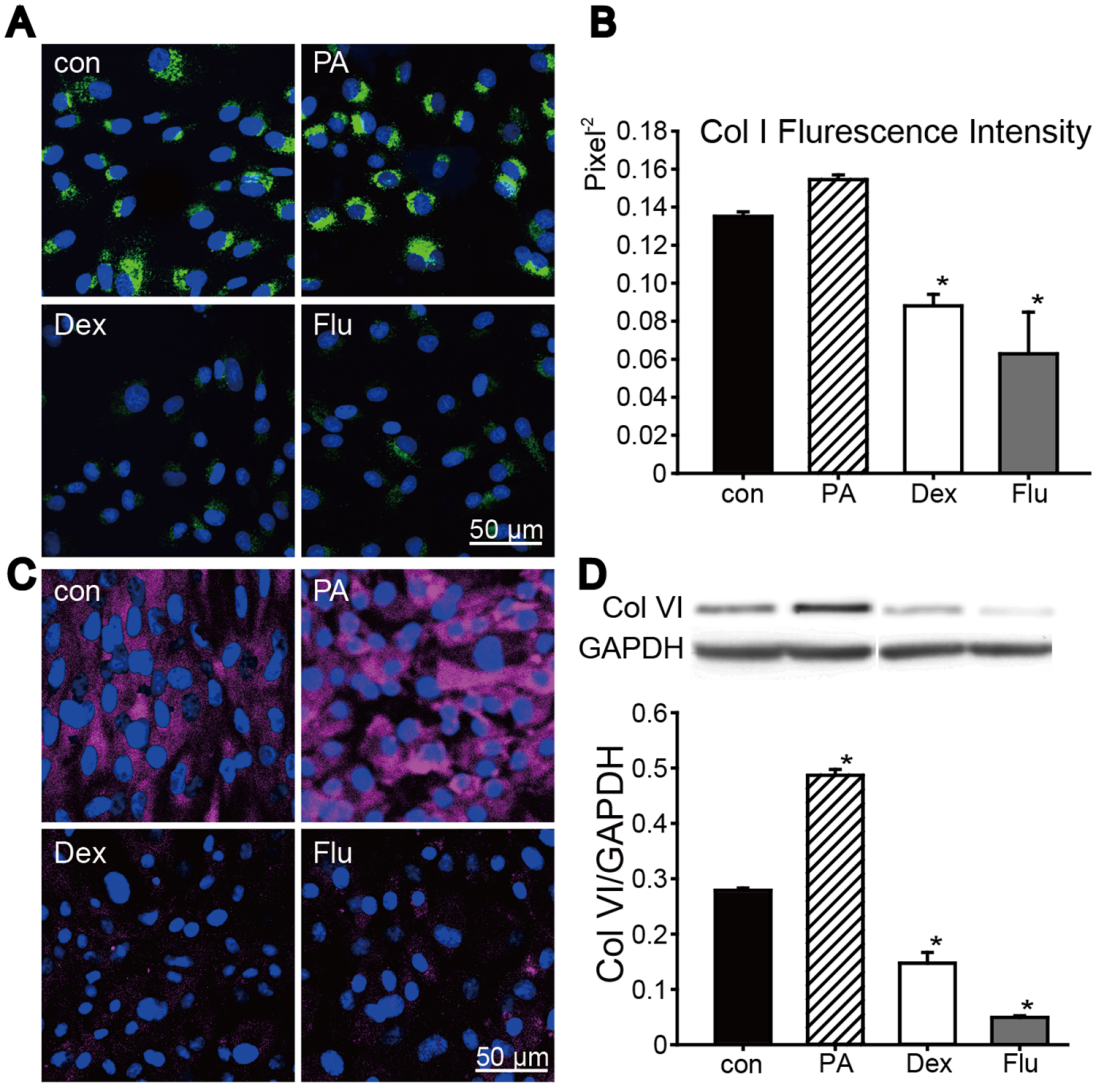
Corticosteroids effects on Col I and Col VI expression. Immunofluorescence staining was carried out on control and corticosteroids-treated keratocytes after 48 h treatment. (A, B) Staining result of Col I (green) was shown, and corresponding fluorescence intensity in each group was also quantified. (C, D) Col VI immunofluorescence staining results were shown (red), and the result was further examined by western blot and corresponding grey scale scanning. Slides were mounted with Hoechst 33528. Data were expressed in mean ± SD, analyzed by one way ANOVA, *p<0.05 versus control group (n = 3).

In normal condition, the expressions of Col I and Col VI were varied among the corticosteroid groups (Fig 2). Immunofluorescence staining results of Col I showed (Fig 2A and 2B) no significant difference between control group and PA group, but Col I expression were significantly weaken in Dex and Flu groups. Additionally, the expression of Col VI was also tested. Col VI immunofluorescence staining showed Col VI expression in PA group was similar to control group, while Dex and Flu significantly decreased Col VI expression. And corresponding results were seen in Col VI western blotting examination (Fig 2D). To conclude, in normal condition, PA showed no suppression effect on Col I and Col VI expression while Dex and Flu significantly decreased their expression.

#### No inflammatory effects were observed in corticosteroids treated keratocytes

**Fig 3:**
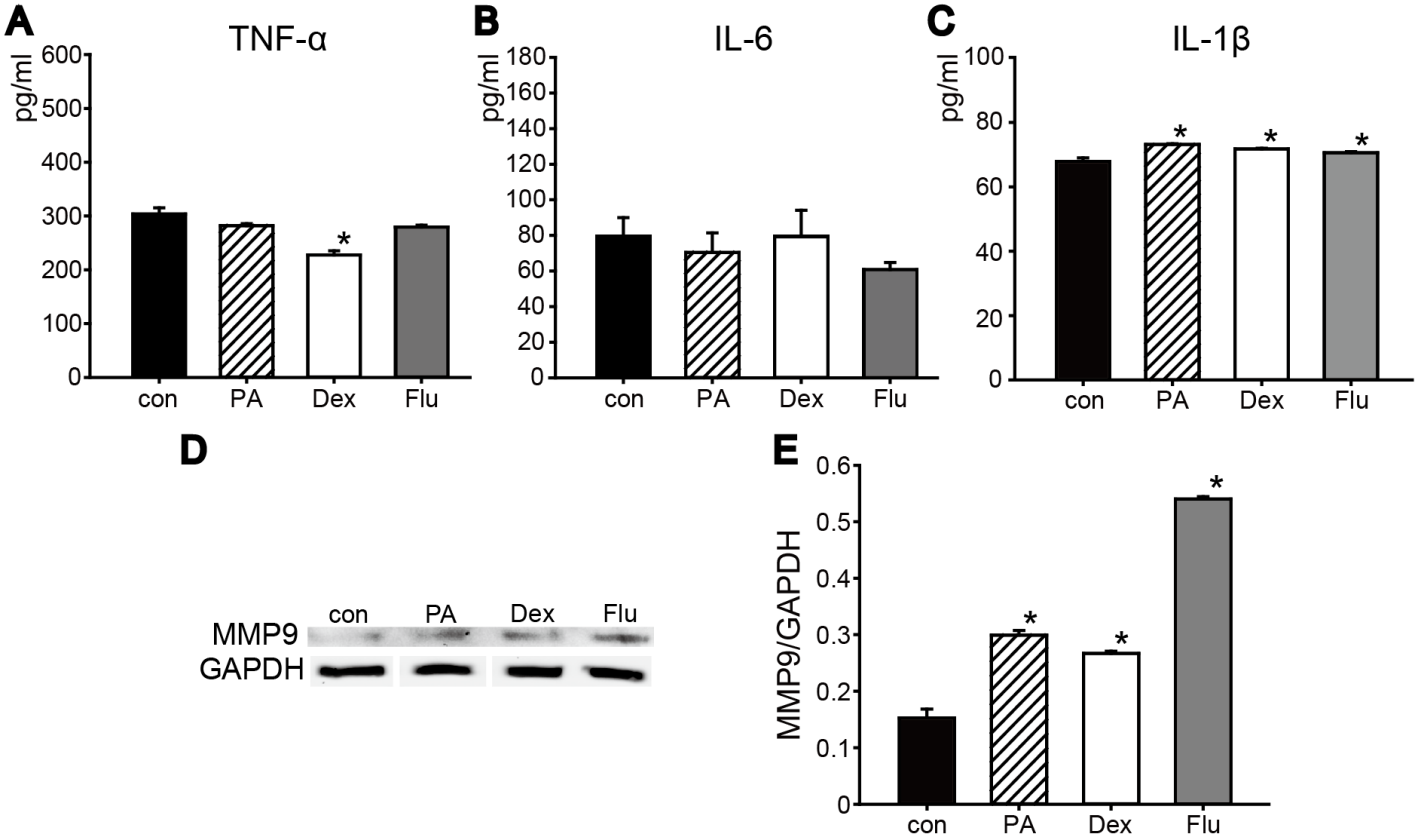
Pro-inflammatory cytokines levels and MMP9 secretion of keratocytes treated with corticosteroids. Keratocytes were grown under normal culture conditions and treated with corticosteroids for 48 h. The levels of TNF-α (A), IL-6 (B) and IL-1β (C) were evaluated after 48 h in culture. (D) MMP9 secretion was tested by Western blot, and quantified result was shown below. *p<0.05 versus control group (n = 3).

Under normal condition, TNF-α, IL-6 and IL-1β levels were tested to further evaluate the effect of PA, Dex, and Flu on corneal keratocytes (Fig 3). TNF-α level of PA group (282±3.85 pg/ml) and Flu group (279.28±3.85 pg/ml) were similar to the control group (303.76±11.54 pg/ml), and a significant decrease was observed inDex group (227.6±7.69 pg/ml) (Fig 3A). There were no significant differences of IL-6 levels among control (79.65±10.34 pg/ml), PA (70.44±10.95 pg/ml), Dex (79.66±14.48 pg/ml), Flu (60.73±4.02 pg/ml) groups under normal condition (Fig 3B). Moreover, similar to IL-6, IL-1β secretion level of keratocytes was even among control group and three corticosteroid groups after 48 h treatments (Fig 3C). These results indicated that in normal condition, keratocytes underwent short-term corticosteroids treatments would not cause inflammatory response *in vitro*. Additionally, analyzed by western blotting, MMP9 level in corticosteroids groups showed a significant increase compared to the control, and Flu had the most significant influence on keratocytes MMP9 expression (Fig 3D).

### Effects of corticosteroids on LPS-treated keratocytes cell model

#### Corticosteroids slightly influenced LPS-keratocytes proliferation or migration

**Fig 4:**
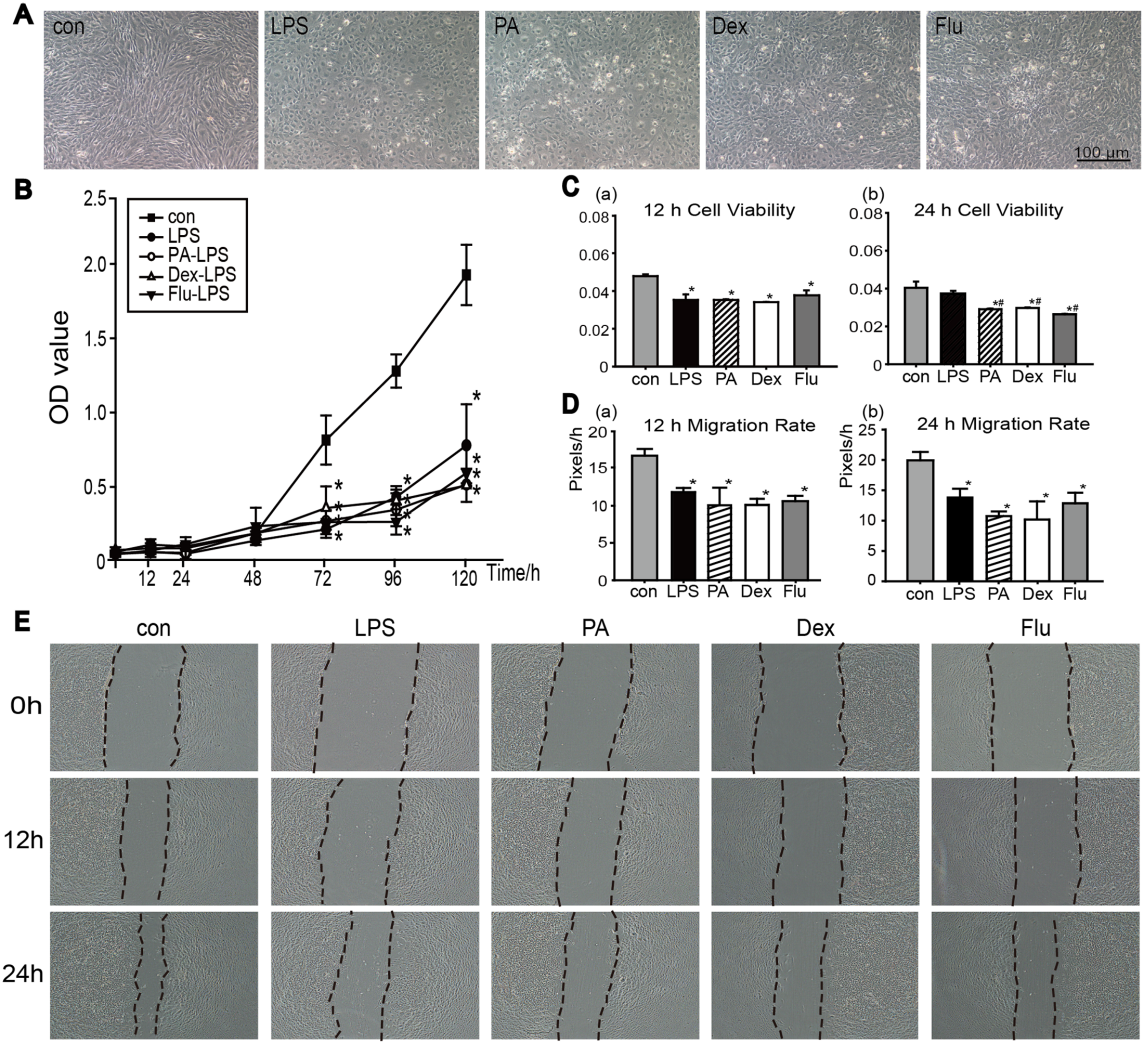
Corticosteroids effects on keratocytes under inflammatory culture conditions. (A) Morphological results at the end of 5-d cell proliferation assay. (B) Proliferation in each group was determined by CCK8 assayed at 12 h, and every 24 h in 5 days. (C) Short-term cell viabilities in each group were tested by CCK8 at 12 h and 24 h. (D) The migration rates of keratocytes at 12 h (a) and 24 h (b) were evaluated by Image Pro-Plus software (Media Cybernetics) using the following formula: (0 h distance—set time distance)/ time (Pixel/h). (E)Migration ability of keratocytes in normal condition with corticosteroid treatments at 0 h, 12 h and 24 h. Data were analyzed by one way ANOVA, *p<0.05 versus control group, # *p*<0.05 versus LPS group (n = 3).

Treated with LPS, cell morphologies changed into polygon shape, and no further obvious differences were seen when the cells received the additional corticosteroid treatments (Fig 4A). The growth rate of all LPS induced groups significantly decreased compared with control group. No significant difference of cell proliferation was observed between LPS group and three corticosteroid-LPS groups during the 5-d growth curve examination (Fig 4B). Moreover, 12 h cell viability assay showed significant decrease in four LPS groups compared to the control group, but no differences were seen between LPS group and three corticosteroid-LPS groups (Fig 4C(a)). However, the result showed significant decrease of cell viabilities in corticosteroid groups at 24 h compared to the LPS group (Fig 4C(b)).

An *in vitro* cell migration assay was performed to determine the migration abilities of keratocytes under inflammation condition (Fig 4D). Migration rates of cells with LPS and corticosteroids treatments showed significant decrease both at 12 h (a) and 24 h (b) (*p*<0.05, n = 3). The suppression effect on keratocytes migration could be easily observed during the test, but additional use of corticosteroids had little effects on keratocytes migration during the early 12 h, which later slightly decreased the migration rate compared to LPS group. Representative images of the width of scratch at 0 h, 12 h and 24 h were shown in Fig 4E.

#### PA and Dex maintained Col VI expression without affecting Col I expression in LPS-keratocytes cell model

**Fig 5:**
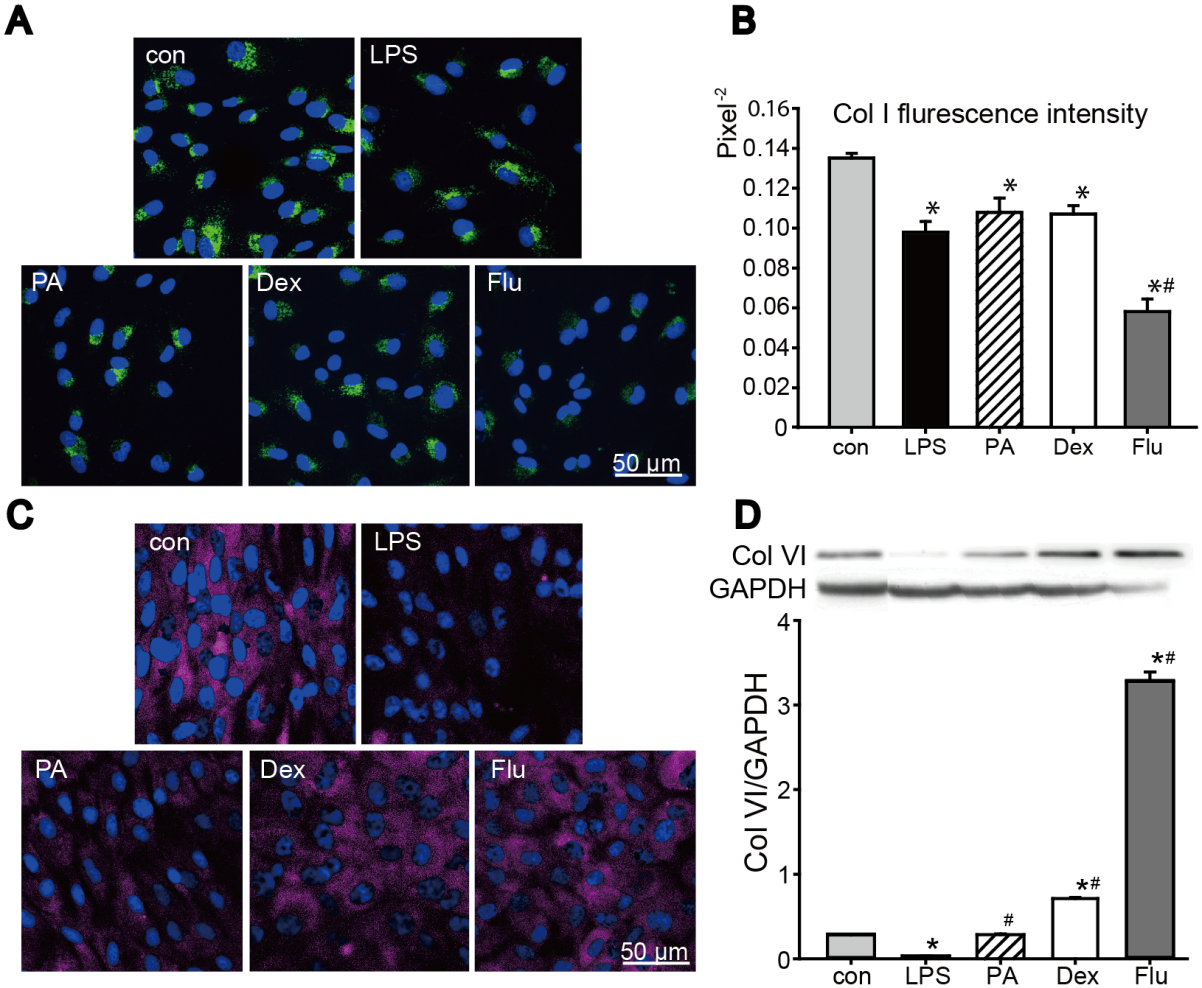
Corticosteroids effects on Col I, VI secretions under inflammatory condition. (A, C)The control and LPS keratocytes cells with PA, Dex, or Flu treatments were stained with the Col I (green), VI (red) marker. (B) Quantified result of Col I immunofluorescence. (D) Col VI expressions by Western blotting and its quantification results. Slides were mounted with Hoechst 33528. Data were expressed in mean ± SD, analyzed by one way ANOVA, *p<0.05 versus control group, #*p*<0.05 versus LPS group (n = 3).

The expression of Col I and Col VI in LPS-indeuced inflammatory keratocyte cell model was tested (Fig 5). As the immunofluorescence staining result and fluorescence intensities showed, Col I expression were obviously weaker in 4 LPS-groups compared to control group (Fig 5A and 5B). No differences of Col I expression were observed between LPS-group and three corticosteroid-LPS groups. As for Col VI, it was clear that collagen secretion in corticorsteroids-LPS groups were higher than that in LPS group. Additionally, the expression of Col VI in PA-LPS group and Dex-LPS group was similar to control group. In Flu-LPS group, Col I level was the least while the expression level of Col VI was the highest among three corticosteroid-LPS groups, and even higher than the control (Fig 5C and 5D).

#### Corticosteroids decreased pro-inflammatory cytokines level in LPS-induced keratocytes cell model

**Fig 6:**
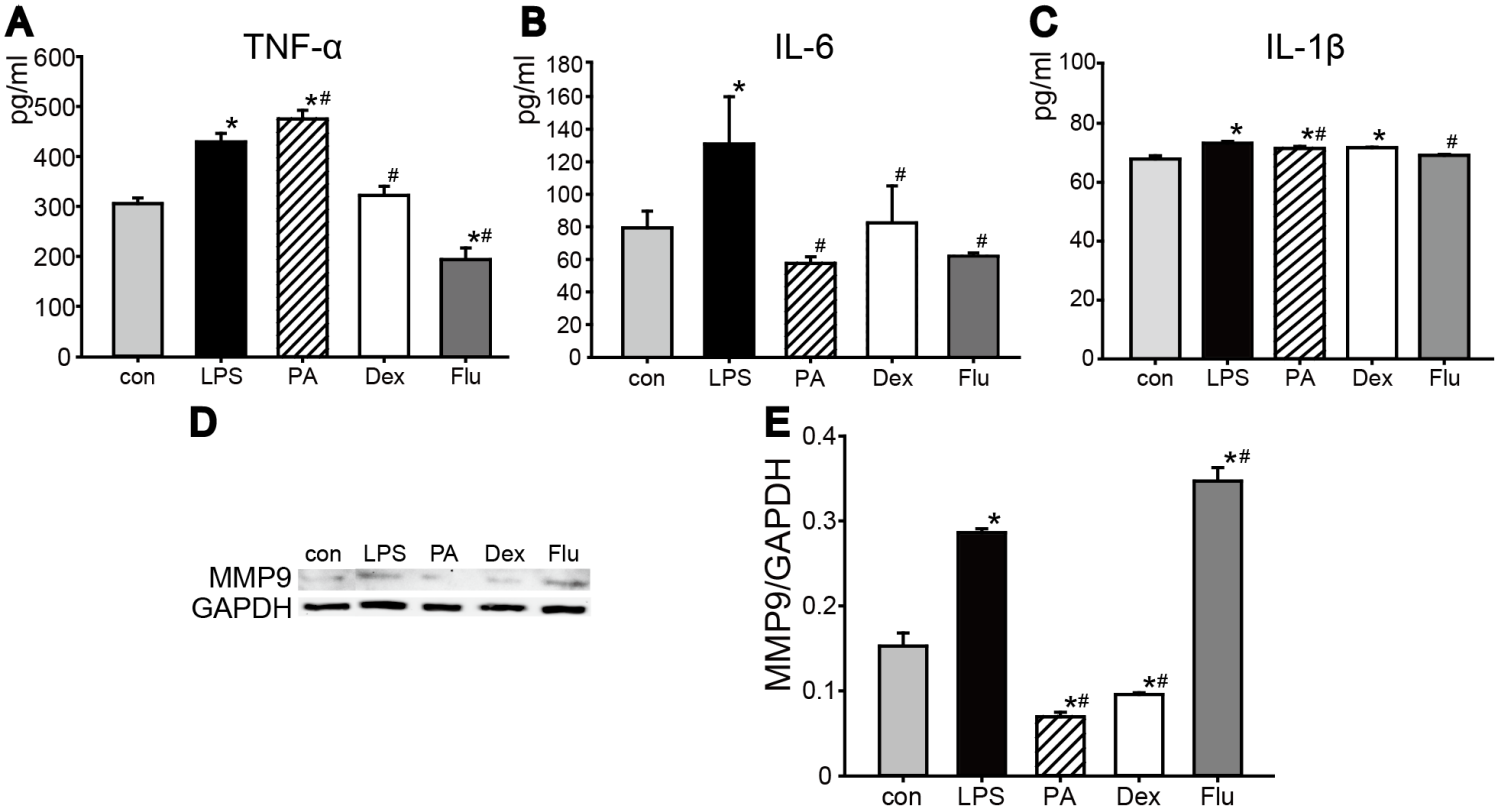
Effect of corticosteroids on the release of pro-inflammatory cytokines and MMP9 in LPS pretreated keratocytes. Keratocytes were treated with 10 μg/ml LPS to induce an inflammatory response. Corticosteroid-LPS groups were added LPS and corticosteroids at the same time to see corticosertoids’ effects on keratocytes under inflammatory condition. The levels of TNF-α (A), IL-6 (B) and IL-1β (C) were evaluated after 48 h in culture. (D)Western blot result and corresponding grey value scanning of MMP9 expression was shown. *p<0.05 versus control group, #*p*<0.05 versus LPS group (n = 3).

To establish an *in vitro* keratitis cell model, keratocytes were treated with LPS. TNF-α, IL-6 and IL-1β levels significantly increased compared to the control (Fig 6A, 6B and 6C). After treated with PA, Dex or Flu with LPS for 48 h, great differences were seen among 3 groups. Compared to the LPS group, the level of TNF-α obviously decreased with Dex (323.2±18.67 pg/ml) and Flu (194.96±23.08 pg/ml), while it significantly increased with PA (476.48±17.31 pg/ml) (Fig 6A). Besides, IL-6 levels in corticosteroid-LPS groups significantly decreased compared to the LPS group (131.05±29.15 pg/ml), among them, PA-LPS (57.73±4.13 pg/ml) and Flu-LPS (62.11±2.06 pg/ml) group were the lowest (Fig 6B). However, little changes could be observed on IL-1β expression and IL-1β level was relatively low even in inflammatory condition (Fig 6C). MMP9 expression was also examined, and quantitative result showed that its expression increased after treated with LPS for 48 h (Fig 5D). However, PA and Dex significantly suppressed this trend, while Flu-LPS group contributed to a much higher expression of MMP9 compared to LPS group.

## Discussion

Keratitis would lead to corneal stroma damage and subsequent scarring formation, which is one of the most serious visual damaging diseases. Although corticosteroids are regarded as common anti-inflammatory eye drops, there is controversy on whether corticosteroids are proper for infectious keratitis treatment at the early stage of keratitis. According to American Academy of Ophthalmology (AAO) guidance in 1980,[13] corticosteroids may suppress inflammation, corneal scarring and associated visual loss, and their suppression effect on collagen secretion was thought to be a major side effect. Moreover, many recent studies showed that using corticosteroids eye drops for acanthamoeba keratitis (AK)[14], fungal keratitis[15] and bacterial keratitis treatments[7, 9] showed better outcomes. Therefore, this study focused on the effect of corticosteroids in cornea healing, anti-inflammation and collagen secretion in both normal and keratitis condition.

In the present study, the effect of corticosteroids on keratocytes in normal condition was studied. The results revealed that corticosteroids groups showed no statistic difference in proliferation with the control group in the early 48 h. Moreover, there were no significant differences of TNF-α, IL-6 and IL-1β level among corticosteroids groups and the control, indicating corticosteroids would not cause cell inflammatory response in normal condition. Although slightly suppression of cell migration and collagen expression were observed, these cell activities did not change the proliferation rate after 48 h culture. Specially, PA group came the nearest to control group above all the experiments in normal condition. These results taken together demonstrated that the use of corticosteroids influenced keratocytes proliferation without leading to an inflammatory in normal condition.

To monitor the anti-inflammatory effect of corticosteroids *in vitro*, an effective inflammatory cell model is required. Many different methods had been used to establish inflammatory cell model *in vitro*, such as co-culture with macrophages,[16] adding inflammatory cytokines like TNF-α or IL-10 in culture medium.[1, 2] As the main component of endotoxin, singly incubation with LPS was also a feasible and usual method for cell model establishment. In our present study, we used 10 μg/ml LPS to induce a clear and strong inflammatory response. As shown in Fig 4, cell migration rate of LPS group was suppressed obviously at 12 h. Similar to other studies, we observed that, the level of pro-inflammatory cytokines such as TNF-α, and IL-6 increased immediately in a short period of time (approximately 2 hours later, data not shown in the manuscript) after adding LPS.[17] IL-1β level was relatively low even if treated with corticosteroids or LPS, indicating that IL-1β secretion might not participate in regulating inflammatory keratocytes. As collagen degradation was one of the main phenomena during keratitis, LPS uppressed both Col I and Col VI secretions in the present study. Significant increase of MMP9 level after LPS incubation was observed. Since inflammation and collagen degradation are the main symptoms during keratitis, these results together demonstrated that single use of 10 μg/ml LPS successfully and effectively built an inflammatory keratitis cell model *in vitro* in the present study.

The sparse-distributed keratocytes in corneal stroma is greatly related to corneal transparency. Normally quiescent in stroma, though, this type of cells can response rapidly and transform into repair phenotypes during injury.[18, 19] However, constant inflammation caused by bacterial infection in wound area would result in the release of pro-inflammatory factors like TNF-α, IL-1 and IL-6[17], which would lead to cel necrosis. With the decline of keratocytes number, cell viability and collagen expression would be suppressed. In the present study, keratocytes proliferation and migration were greatly suppressed under LPS induction, but no such effect on proliferation in the corticosteroids groups under inflammatory condition was observed. Moreover, better migration was observed in the first 6h in PA-LPS group. Keratocytes proliferation and migration are not the main mechanism for cornea repair and over proliferation of keratocytes could lead to corneal opacification. Therefore, it is acceptable that corticosteroids protected the basic function of keratocytes in inflammatory condition mainly through its anti-inflammatory effect, which is crucial for preventing cell necrosis as well as maintaining collagen secretion and structure during corneal wound healing process. It was interesting noticing that corticosteroid groups significantly decreased pro-inflammatory cytokines level *in vitro*, especially TNF-α and IL-6. These results indicated an anti-inflammatory effect of corticosteroids on LPS-induced keratocytes without affecting proliferation or migration activities.

Maintaining expression and structures of major collagen rather than proliferation of keracytotes plays key factors on preventing corneal stroma melting. Patrick et al reported that calf corneal stroma contained approximately 75% Col I, 8% Col V, and 17% Col VI.[20] These collagens are crucial in forming and maintaining the space of collagenous fiber, which is important for keeping transparency.[21–23] Col I serves as a support of corneal stroma, and the interrelationship between Col I and Col V maintains the mechanical strain and regulates fibril diameter.[23, 24] In normal condition, corticosteroids would slightly suppress keratocyte cell viability, which would influence Col I and Col VI expression. Moreover, LPS-induced inflammation had a heavier suppressive effect on collagen expression. However, this suppressive effect was mitigated by adding corticosteroids into LPS group. The anti-inflammatory effect of corticosteroids resulted in protecting the capability of collagen expression. The Col I levels in both PA-LPS and Dex-LPS groups were higher than LPS group, though they were still lower than control. This result indicates that corticosteroids could protect collagen expression through its anti-inflammatory effect. Col VI is important in sustaining normal collagen structure; however, its expression mainly increases during wound healing process. The orderly interaction of Col VI and glycoconjugates produce a normal and optically useful stroma.[25–27] In Dex-LPS and Flu-LPS groups, an upward trend of Col VI expression could be clearly observed. This trend would be benefit for maintaining corneal transparency and structure, so that corneal damage caused by inflammation could be reduced. So we hypothesize that corticosteroids could protect Col I as well as improve the expression of Col VI under inflammatory environment, indicating that using corticosteroids at the early stage of inflammation would contribute benefit to wound healing, maintaining corneal transparency and the reconstruction of collagen.

Matrix metalloproteases (MMPs) are a group of specialized proteolytic enzymes that may degrade the complex molecules constituting the extracellular matrix in certain conditions.[28–30] Among them, MMP1, MMP3 and MMP9 are mostly discussed. 92 kDa gelatinase B (MMP9) is the primary matrix-degrading enzymes and plays an important role in corneal wound healing, especially in ECM remodeling. Ladwig et al. reported that within the wound healing process, the level of MMP9 decreased in chronic wound fluid.[31] Thus, suppressing the upward trend of MMP9 may improve the poor wound healing condition of keratitis. In the present study, we considered MMP9 as our tool to evaluate the degree of anti-inflammatory effect caused by corticosteroids. The result showed that PA and Dex greatly suppressed MMP9 level in inflammatory cell model while increased Col I expression Therefore, it is reasonable to suggest that using corticosteroids, especially PA and Dex, at the early stage of keratitis could maintain the main collagen component, Col I and Col VI, expression in stroma and reduced its degradation while decreasing MMP9 expression.

The effects of three different corticosteroids groups in inflammatory cell model were various. PA, Dex and Flu groups showed similar influence on keratocytes proliferation and migration. Pro-inflammatory cytokines like IL-1, IL-6 and TNF-α produces by keratocytes is also an important factor causing corneal ulcer. As for anti-inflammatory effect, Dex and Flu groups significantly decreased TNF-α, IL-6 level, while PA significantly decreased IL-6 level in LPS-induced keratocytes. As maintaining collagen expression is one of the most important factors, PA maintained both Col I and Col VI expressions in normal as well as inflammatory conditions, while Flu showed unstable effects. Additionally, the expressions of MMP9 were even in PA and Dex groups, and it was upward by Flu in both conditions. Therefore, it is suggested that PA could be an effective drug candidate for keratitis treatment at the early stage of keratitis.

To conclude, slightly suppression of corticosteroids on keratocytes proliferation and migration were observed, but corticosteroids showed anti-inflammatory effects as well as maintaining Col I and Col VI expressions in LPS-induced keratocytes. Among three corticosteroids, PA showed the least influence on keratocytes in normal condition and it significantly decreased IL-6 level and maintained collagen synthesis in LPS-induced keratocytes, which is suggested as an anti-inflammatory drug treatment at the early stage of keratitis.
